# Supplementary material for: Structural insight into guanylyl cyclase receptor hijacking of the kinase–Hsp90 regulatory mechanism
Source: bioRxiv. 2023 Feb 14:2023.02.14.528495. Preprint. [Version 1] doi: 10.1101/2023.02.14.528495 (PMC9948968; doi:10.1101/2023.02.14.528495)
Supplement: Supplementary file 1 [file NIHPP2023.02.14.528495v1-supplement-1.pdf]

bioRxiv preprint doi: https://doi.org/10.1101/2023.02.14.528495; this version posted February 14, 2023. The copyright holder for this preprint (which was not certified by peer review) is the author/funder, who has granted bioRxiv a license to display the preprint in perpetuity. It is made available under aCC-BY 4.0 International license.

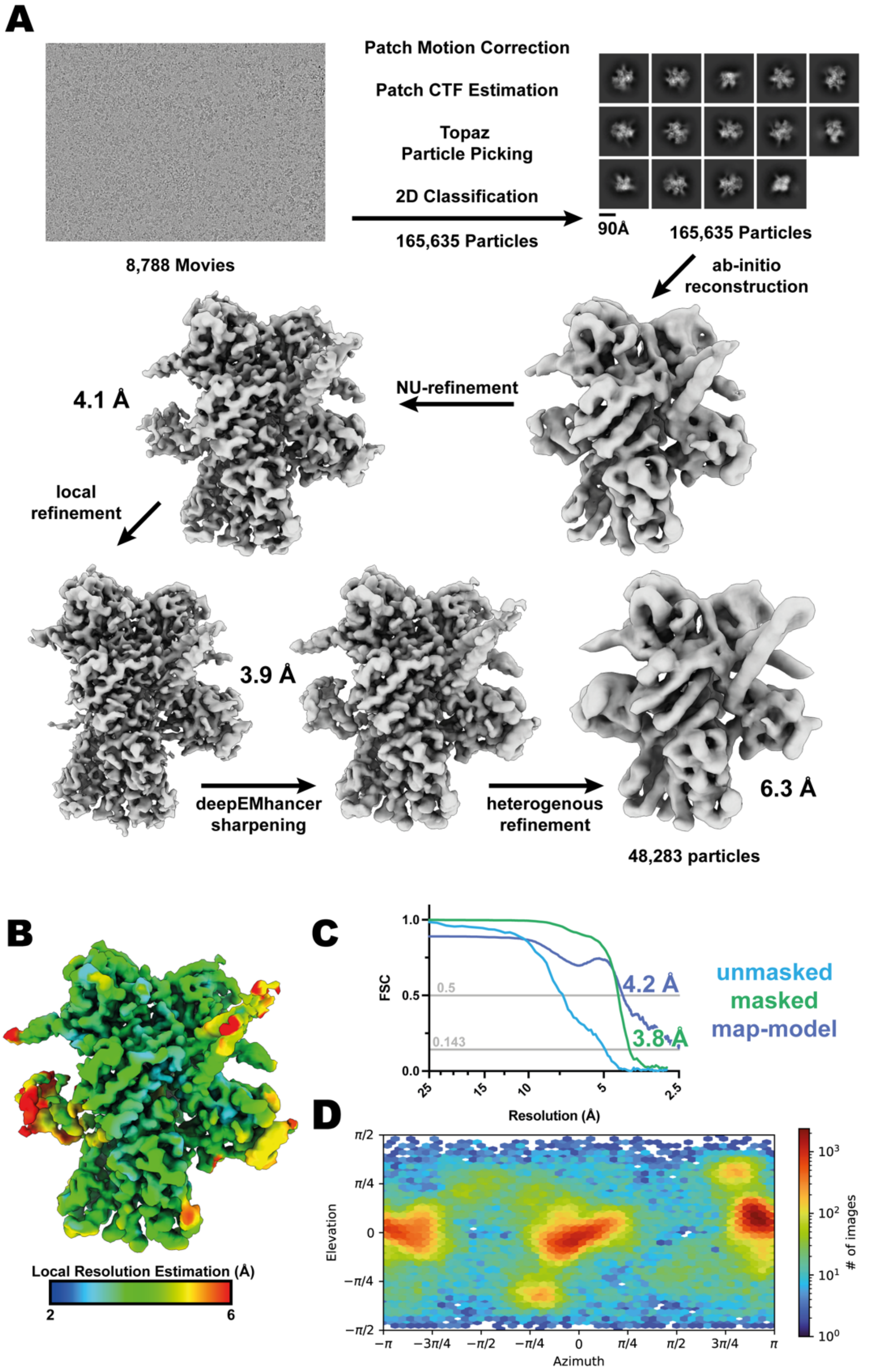

Supplemental Figure 1. GC-C–Hsp90–Cdc37 complex cryoEM data processing. (**A**) Workflow for cryoEM data processing. Representative micrograph, reference free 2D averages, and cryoEM maps at the various stages of processing. (**B**) Local resolution estimation of the finalised cryoEM map. (**C**) FSC curve of the reconstruction using gold-standard refinement calculated from unmasked and masked half maps. Map-model FSC curve. (**D**) Orientational distribution of the reconstruction.

bioRxiv preprint doi: https://doi.org/10.1101/2023.02.14.528495; this version posted February 14, 2023. The copyright holder for this preprint (which was not certified by peer review) is the author/funder, who has granted bioRxiv a license to display the preprint in perpetuity. It is made available under aCC-BY 4.0 International license.

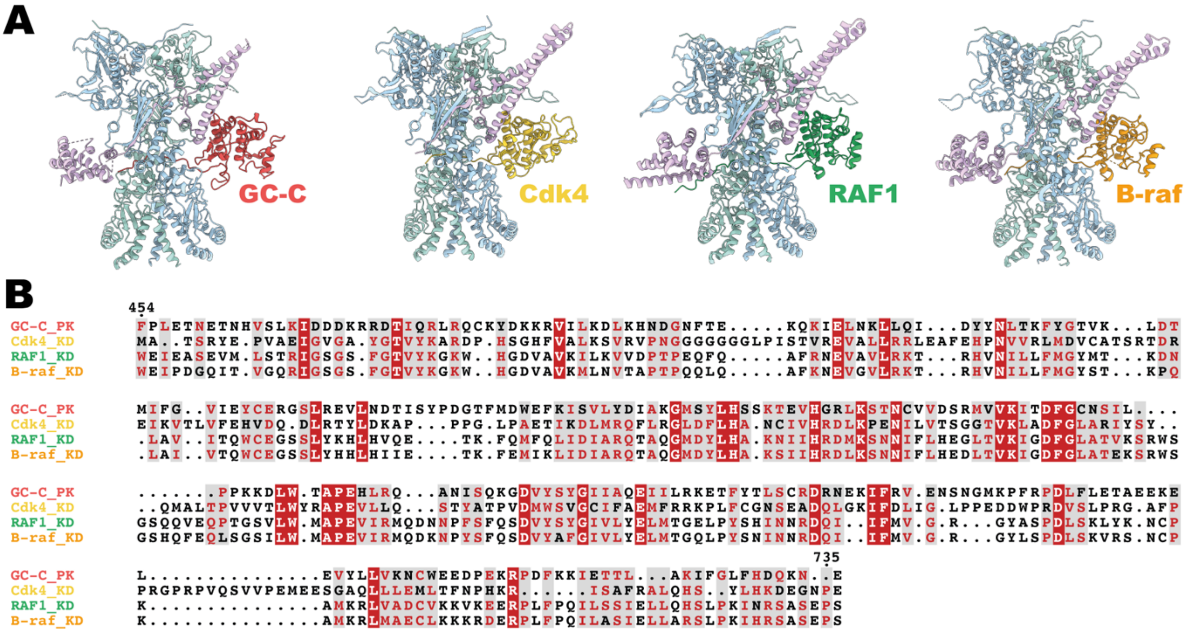

Supplemental Figure 2. Conservation of Cdc37 mediated Hsp90 regulation. (**A**) Ribbon representation of a models of client–Hsp90β–Cdc37 complexes. GC-C is colored in red, Cdk4 in yellow (5FWK), RAF1 in green (7Z37), B-raf in orange (7ZR0), Hsp90β in light blue and teal, and Cdc37 in light purple. (**B**) A sequence alignment of the pseudokinase domain of GC-C and the kinase domains of Cdk4, RAF1, and B-raf.

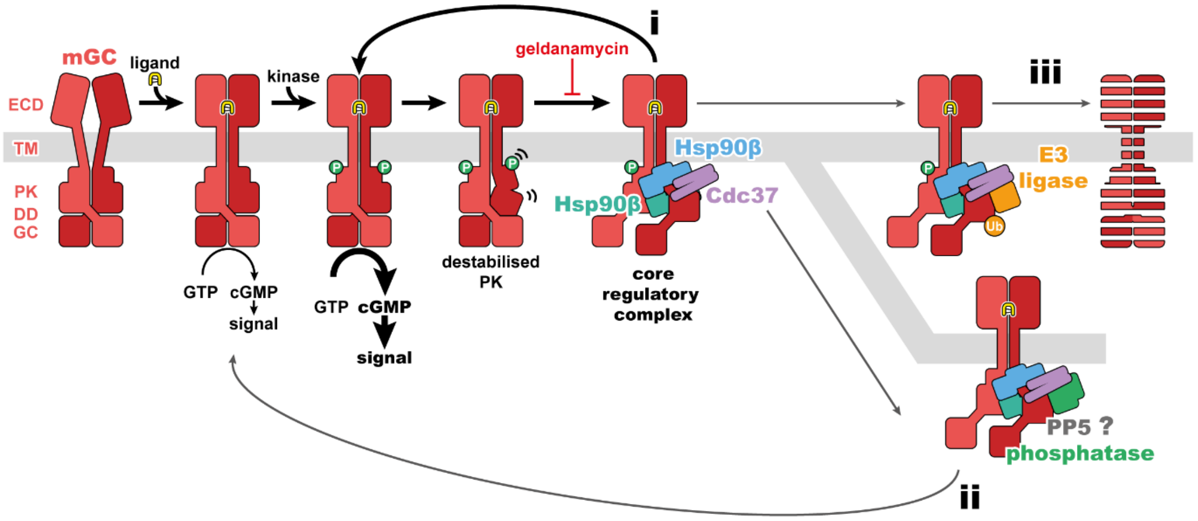

Supplemental Figure 3. Regulatory mechanisms for mGC activity. A schematic of mGC ligand induced activity, phosphorylation, and destabilization, leading to formation of the mGC–Hsp90–Cdc37 complex structurally characterized in this work. This core regulatory complex would then lead to refolding of the PK and reactivation of the receptor (**i**), recruitment of PP5 and dephosphorylation of the receptor (**ii**), or recruitment of E3 ligases and removal of the receptor (**iii**). An mGC is depicted in red, ligand in yellow, Hsp90 in blue and teal, Cdc37 in purple, a phosphatase in green, and an E3 ligase in orange.

bioRxiv preprint doi: https://doi.org/10.1101/2023.02.14.528495; this version posted February 14, 2023. The copyright holder for this preprint (which was not certified by peer review) is the author/funder, who has granted bioRxiv a license to display the preprint in perpetuity. It is made available under aCC-BY 4.0 International license.

Supplementary Table 1. CryoEM data collection, refinement, and validation statistics.

<table>
<tr><th></th><th>GC-C–Hsp90–Cdc37 complex
PDB 8FX4
EMD-29523</th><th>GC-C–Hsp90–Cdc37 complex
with DD density</th></tr>
<tr><td>Data collection and processing</td><td></td><td></td></tr>
<tr><td>Magnification</td><td colspan="2">45,000</td></tr>
<tr><td>Voltage (keV)</td><td colspan="2">200</td></tr>
<tr><td>Electron exposure (e^-/Å^2)</td><td colspan="2">58.8</td></tr>
<tr><td>Defocus range (μm)</td><td colspan="2">0.8 to 2.0</td></tr>
<tr><td>Pixel size (Å)</td><td colspan="2">0.9273</td></tr>
<tr><td>Symmetry imposed</td><td colspan="2">C1</td></tr>
<tr><td>Final particle images</td><td>165,635</td><td>48,283</td></tr>
<tr><td>Map resolution FSC threshold</td><td colspan="2">0.143</td></tr>
<tr><td>Map resolution (Å)</td><td>3.9</td><td>6.3</td></tr>
<tr><td>Refinement</td><td></td><td></td></tr>
<tr><td>Initial model used (PDB)</td><td>5FWK, 7ZR5, AlphaFold</td><td></td></tr>
<tr><td>Model resolution FSC threshold (Å)</td><td>0.5</td><td></td></tr>
<tr><td>Model resolution (Å)</td><td>4.2</td><td></td></tr>
<tr><td>Model Composition</td><td></td><td></td></tr>
<tr><td>Non-hydrogen atoms</td><td>13,478</td><td></td></tr>
<tr><td>Protein residues</td><td>1,654</td><td></td></tr>
<tr><td>Ligands</td><td>2</td><td></td></tr>
<tr><td>B-factors (Å^2)</td><td></td><td></td></tr>
<tr><td>Protein</td><td>119.49</td><td></td></tr>
<tr><td>Ligand</td><td>102.85</td><td></td></tr>
<tr><td>R.m.s. deviations</td><td></td><td></td></tr>
<tr><td>Bond lengths (Å)</td><td>0.004</td><td></td></tr>
<tr><td>Bond angles (°)</td><td>0.914</td><td></td></tr>
<tr><td>Validation</td><td></td><td></td></tr>
<tr><td>MolProbity score</td><td>2.14</td><td></td></tr>
<tr><td>Clashscore</td><td>13.88</td><td></td></tr>
<tr><td>Rotamer outliers (%)</td><td>0.67</td><td></td></tr>
<tr><td>Ramachandran plot</td><td></td><td></td></tr>
<tr><td>Favoured (%)</td><td>92.0</td><td></td></tr>
<tr><td>Allowed (%)</td><td>7.6</td><td></td></tr>
<tr><td>Outliers (%)</td><td>0.4</td><td></td></tr>
</table>
